# A whole mitochondria analysis of the Tyrolean Iceman’s leather provides insights into the animal sources of Copper Age clothing

**DOI:** 10.1038/srep31279

**Published:** 2016-08-18

**Authors:** Niall J. O’Sullivan, Matthew D. Teasdale, Valeria Mattiangeli, Frank Maixner, Ron Pinhasi, Daniel G. Bradley, Albert Zink

**Affiliations:** 1 Institute for Mummies and the Iceman, EURAC research, 39100 Bolzano, Italy; 2 School of Archaeology and Earth Institute, University College Dublin, Belfield, Dublin 4, Ireland; 3 Smurfit Institute of Genetics, University of Dublin, Trinity College, Dublin 2, Ireland

## Abstract

The attire of the Tyrolean Iceman, a 5,300-year-old natural mummy from the Ötzal Italian Alps, provides a surviving example of ancient manufacturing technologies. Research into his garments has however, been limited by ambiguity surrounding their source species. Here we present a targeted enrichment and sequencing of full mitochondrial genomes sampled from his clothes and quiver, which elucidates the species of production for nine fragments. Results indicate that the majority of the samples originate from domestic ungulate species (cattle, sheep and goat), whose recovered haplogroups are now at high frequency in today’s domestic populations. Intriguingly, the hat and quiver samples were produced from wild species, brown bear and roe deer respectively. Combined, these results suggest that Copper Age populations made considered choices of clothing material from both the wild and domestic populations available to them. Moreover, these results show the potential for the recovery of complete mitochondrial genomes from degraded prehistoric artefacts.

The Tyrolean Iceman, a 5,300-year-old (Copper Age) natural mummy, discovered in the Italian Ötztal Alps in 1991 provides a direct archaeological link to prehistoric Europe. Two decades of analysis concerning this individual have provided insights into ancestry, diet, tools, lifestyle, health and attire^1^,^2^,^3^. Despite multiple studies and their relatively good preservation, ambiguity still persists as to the species of origin for the majority of the Iceman’s clothes^4^,^5^. A more complete characterisation of his garments is therefore required, if we are to maximise the information provided by these artefacts and gain further insights into the choice of raw material from which Copper Age populations manufactured their clothes.

Preserved leathers provide rare and valuable information into how ancient populations utilized the secondary products of animal husbandry^6^,^7^. To date biomolecular research on the Iceman’s leather has been confounded by the relatively high decomposition of the material^3^. The structural features of the leather and fur necessary for microscopic identification, such as grain pattern, are either damaged or absent from the clothing^4^. Proteomic and microscopic analyses have managed to some extent to overcome these limitations^4^,^5^, allowing for a species level resolution of some material, but ambiguity still remains in regard to others. For instance, it has been reported that a peptide mass fingerprinting (PMFs) analysis of the clothes, based on keratins and collagen recovered from the ancient leather, provided insufficient data to differentiate between closely related species such as goat and sheep^6^,^7^. Analysis of genetic data is therefore critical if we are to distinguish leather sourced from domesticated species from those produced from closely related wild relatives^8^. Furthermore, populations of the same domestic species can be differentiated by genetic markers in the mitogenome (haplogroups) that indicate independent domestications and human-mediated migrations, observing these haplogroups can highlight the genetic origin of the population used in clothing manufacture^9^.

Previous PCR work targeting ancient DNA (aDNA) fragments extracted from leather and furs have reported, in most cases, poor endogenous content^10^. This lack of such aDNA (apart from time dependent decomposition) has been attributed to the deterioration and removal of DNA molecules during the manufacturing process, which is thought to involve scraping, exposing to fatty acids and, in some cases, intense heating^11^,^12^. In addition, the ancient tanning process is believed to introduce contamination from handling by humans or by the addition of organic material from other mammalian species^5^. Finally, the restoration process or the freeze-drying of the leather may have leached away identifying biological markers^11^. Recently, PCR based genetic analyses of hair shafts, recovered from more recent excavations of the Iceman find site have succeeded in further characterising the clothes; providing a species identification and even comparative phylogenies with modern populations^13^,^14^. These samples are however, unfortunately not directly associated with particular parts of the Iceman’s clothing, and as such lack this critical piece of information for the evaluation of material usage by Copper Age individuals.

Conventional PCR based sanger sequencing of aDNA is further restricted by low endogenous DNA content, small fragment sizes and the co-extraction of contaminating molecules that are typical of ancient material^15^,^16^. The innovations of high throughput sequencing and targeted enrichment of specific DNA markers have overcome many of the obstacles faced by researchers in the study of DNA extracted from such archaeological materials^17^, and in short have led to the identification and characterisation of a wide range of informative genetic markers from ancient human and animal populations^18^,^19^,^20^,^21^,^22^,^23^,^24^,^25^,^26^,^27^,^28^. The study of ancient animal genetic variation can uncover novel variety or distributions of ancient populations that would not be possible solely from the analysis of modern genetic data, as intensive selective pressure (especially for domestics in the past 200 years^29^) and migrations have shuffled European phylogeography^30^. This has shown that contemporary genetic distributions of European livestock are not necessarily representative of ancient distributions, such as during the Copper Age or Neolithic^31^. In this paper, we report a targeted mitochondrial enrichment of DNA extracted from six articles of the Iceman’s personal effects: shoelace, hat, loincloth, coat, leggings, and quiver. We then use these data to provide a discussion of Copper Age leather production and to investigate the relationship between the ancient animals and their current day populations.

## Results

### DNA enrichment, quality and authentication

The sequencing libraries of the Iceman’s clothes were subjected to both a low coverage shotgun sequencing analysis and mtDNA capture (targeted enrichment), using the Illumina MiSeq platform. To assess the quality of the extracted DNA and to measure the success of the mitochondrial capture, the mapped reads from the shotgun and capture libraries were quality controlled and quantified (Table 1 and Supplementary Table 2). Firstly, the libraries were shotgun sequenced (except for the hat sample, which was only sequenced after enrichment) and the presence of species-specific markers was ascertained. However, shotgun depth of coverage was too low for the majority of samples to reconstruct complete mitogenomes or recover enough informative autosomal markers, as most reads in sequenced libraries (often >99%) were exogenous. Hence, deeper shotgun sequencing to recover complete nuclear or mitochondrial genomes was not economically feasible. To overcome this situation, the samples were enriched for mitochondrial templates, which allowed for a four to 620-fold increase in high-quality unique sequences mapping to the mitochondrial reference. Fold-increase was based upon a down-sampling of captured or shotgun libraries to a similar number of reads depending on the depth of sequencing, after PCR-duplicates were removed. With the exception of the hat, quiver and light coat (0.4x–3.6x mean coverage), there was sufficient coverage to reconstruct complete mitogenomes for six of the libraries (15.5x–110x mean coverage). A positive and unambiguous species assignment for all enriched libraries was made regardless of coverage. For the six highly covered libraries a mapDamage^32^ analysis (Supplementary Fig. 1) of sequence data showed damage patterns indicative of antiquity. However, this authenticity check is only optimal for relatively high coverage data, as the signal to noise ratio was observed to decline with increased coverage. This is shown by the unclear damage patterns of the three low covered libraries (Supplementary Fig. 2). Cross-contamination estimates from multiple animal sources were low (<5%) (Table 1). These values were based on the average number of all mismatches at haplogroup defining haplotypes (Supplementary Table 3). Sequence depth of cover was visualized using qualimap software (bamqc)^33^ and displayed no disproportionately low coverage that could disrupt consensus calls (Supplementary Figs 11–19) for the six highly covered libraries.

### Phylogeny and haplogroup assignment

**Figure 1:**
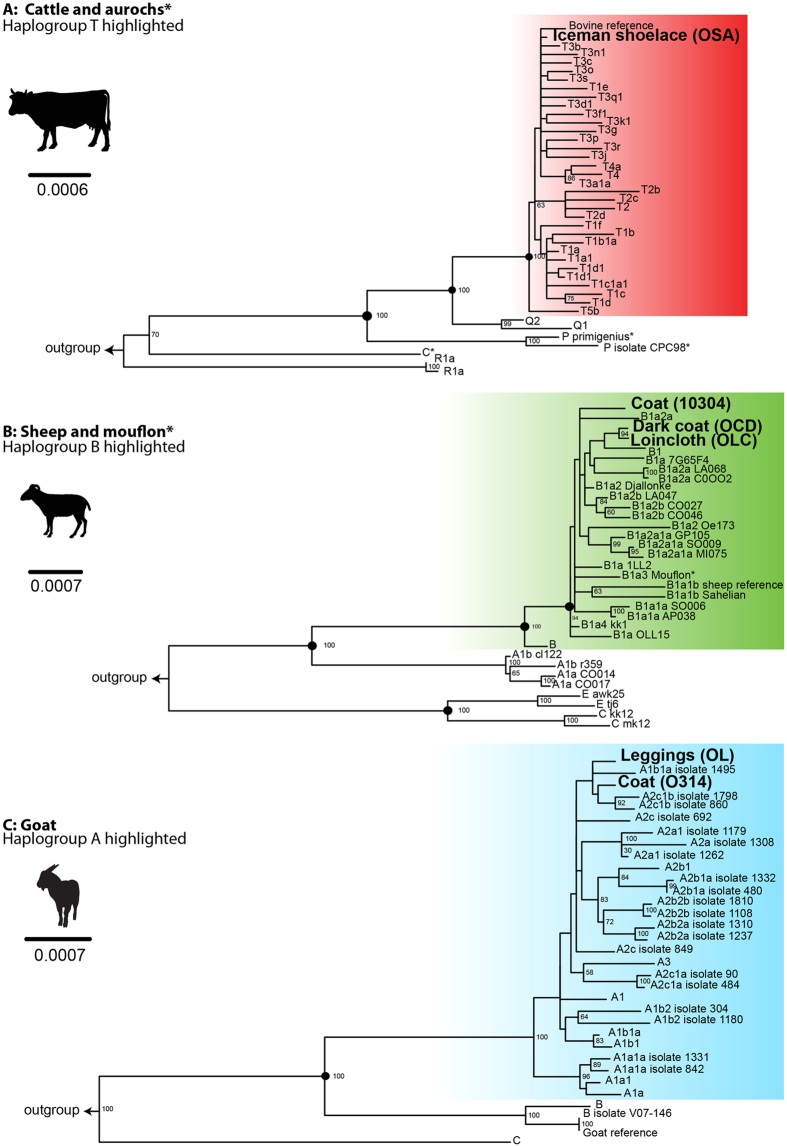
Phylogeny based on fully reconstructed mitogenomes. Asterisks in labels of branches in trees (**A**) and (**B**) correspond to asterisks on respective clade/haplogroup labels where inter-species mono-phylogeny occurs. Outgroups used to root trees have been removed (Supplementary Table 1). Black circles on nodes indicate that there was more than 90% bootstrap support in neighbour joining, maximum parsimony and maximum likelihood topologies. Coloured boxes highlight associated haplogroups of samples. Animal illustrations provided by Matthew Collins (*pers coms*).

**Figure 2:**
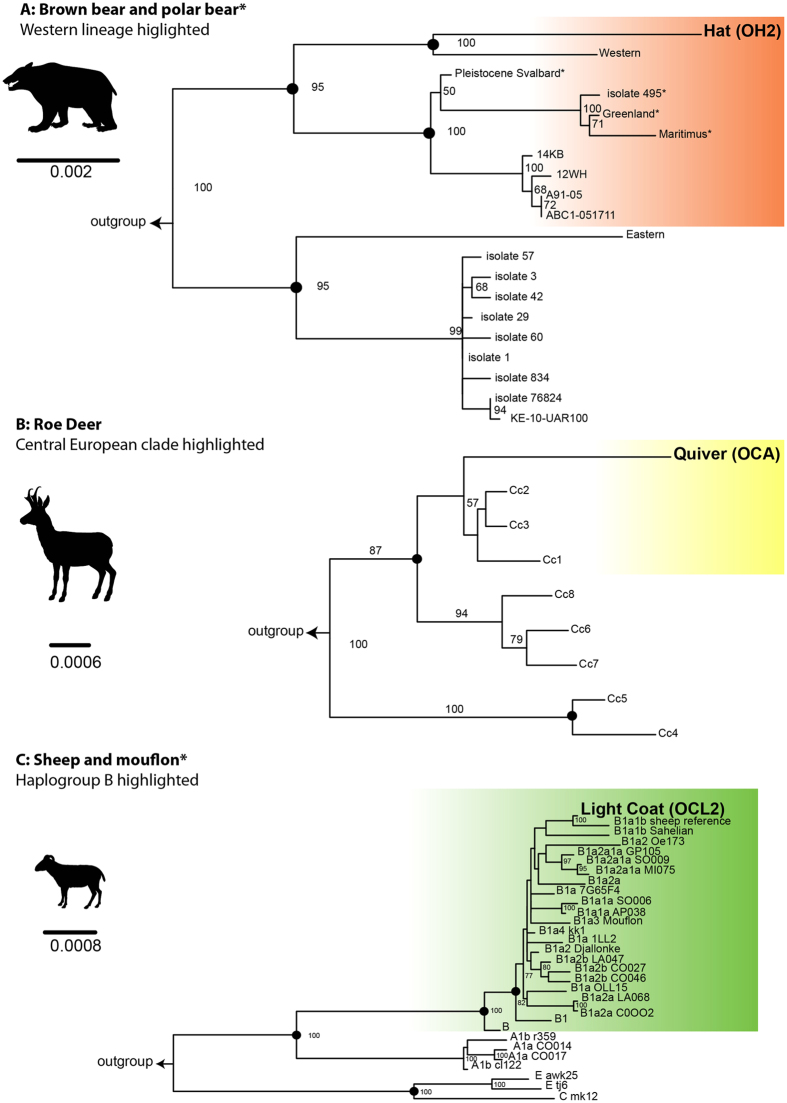
Species phylogeny based on partially reconstructed mitogenomes. Aligned sequences had all gap sites removed with Gblocks, except the sheep light coat (OCL2). Asterisks on branch labels of trees **(A)** and **(C)** correspond to clade/haplogroup names where inter-subspecies mono-phylogeny occur. Outgroups used to root trees have been removed (Supplementary Table 1). Black circles on nodes indicate that there was more than 90% bootstrap support in neighbour joining, maximum parsimony and maximum likelihood topologies. Coloured boxes highlight associated haplogroups of samples. Animal illustrations provided by Matthew Collins (*pers coms*).

The haplogroups and haplotypes of the mitochondrial genomes sequenced from libraries produced from the leather of domestic species were identified with the aid of MitotoolPy^34^ tool and visually inspected using IGVtools^35^ (Table 1). All reported mutations are relative to the reference sequences (Supplementary Table 1 and Supplementary Table 3). Phylogenetic reconstructions of completely and partially reconstructed mitochondria were produced for all libraries (Figs 1 and 2, respectively).

The shoelace clustered to haplogroup T3 taurine lineage of cattle (Fig. 1a). The loincloth, dark fur coat and coat (sample 10304) libraries clustered inside sheep haplogroup B1, closer to modern domestics than mouflon (*Ovis orientalis*) (Fig. 1b). The coat sample (O314), and the leggings were identified as goat haplogroup A (Fig. 1c). There was sufficient mitochondrial genetic diversity observed to show that the materials derived from both sheep and goat came from multiple individuals, there were at least four sheep and two goats used in the manufacture. Goat and sheep sequences clustered closely within their respective haplogroups (Figs 1 and 2). Visualisation and contamination estimates of sequences did not show a co-capture of goat nuclear copies of mitochondrial DNA (numt), which have confounded some reference datasets for goat^36^.

The three low coverage libraries provided sufficient markers for clade or haplogroup assignment, but care is needed in their interpretation as SNP verification was constrained by low coverage. The sheep light coat sample exemplifies this problem, as it had a substantially lower coverage than the other identified domestic mitogenomes. This meant that potential sequence errors could not be removed by applying a majority consensus call alone. Hence, the sheep light coat data was mapped at a higher stringency with BWA aln (argument: -n 0.1) to remove confounding sequences. This method removed the confounding data, on inspection with IGVtools these appeared to be mostly coding lesions caused by relatively high degradation, and produced a more conservative consensus sequence. The resulting phylogeny for the light coat had a shorter branch length but was more realistic, which clustered approximately within sheep haplogroup B1 (Fig. 2c). The light coat phylogeny was based on 15,444 bp (base pairs) retrieved sites out of the complete 16,616 bp reference. The hat was identified as brown bear clustering nearest the European Western lineage (Fig. 2a), based on a reduced dataset of 5,363 bp of retrieved sequence out of a complete genome of 16,870 bp. The quiver was roe deer, which clustered closest to the central European clade (Fig. 2b). The roe deer sequence consisted of 7,662 bp, reconstructed compared to the 16,358 bp long reference mitogenome.

## Discussion

Here we present a comprehensive targeted enrichment and species identification of 5,300-year-old leather from the Iceman’s clothes and quiver (Table 1). Of the nine samples taken, all produced libraries with sufficient data to identify the source species of the material. These were cattle, sheep, goat, brown bear and roe deer. Six libraries had sufficient coverage to fully reconstruct the mitogenome and confidently identify mtDNA haplotypes and reconstruct phylogeny (Fig. 1). Three libraries were of reduced coverage, thereby only partially reconstructing the mitogenomes and allowing for an approximated phylogeny (Fig. 2). Nonetheless, the identified cattle, sheep and goats sequences all fall within the range of mitochondrial genetic variability observed today in modern European domestic populations^36^,^37^,^38^. The mitogenomic genetic variability recovered shows that the sheep and goat leather fragments came from multiple hides. The genetic haplotypes of the wild species both roe deer and brown bear are consistent with present day phylogeography in the Alpine region^39^,^40^.

The Mitochondrial capture analysis allowed for an effective increase in coverage (between a four and 620-fold increase in coverage) and highlighted the effectiveness of NGS assisted with mitochondrial targeted enrichment to retrieve residual endogenous DNA from suboptimal sources such as skin or tanned leather^10^,^41^. Furthermore, our shotgun analysis is the first NGS quantification of the residual endogenous DNA present in archaeological leather. These data show that the endogenous content was highly variable and considerably lower than that of tissue sampled from the Iceman for genomic reconstruction^3^. This result lends support to the theory that tanning, the restoration processes, or both can remove endogenous markers^10^,^11^ and furthermore that the tanning may have exposed the material to a greater degree of bacterial and chemical degradation^7^. Equally, these results could support the hypothesis that soft tissue has a generally weaker retention of endogenous DNA compared to calcified remains^41^. Interestingly, enriched libraries displayed neither co-enrichment from other mammalian species or cross contamination from other animals in the mapped data (Supplementary Figs 2–10, Table 1). Hence, there is little genetic evidence from these samples to suggest that leathers and furs were treated with fat and oil extracted from other animals during the ancient tanning process^11^. Previous studies have however observed residual plant-based oils and fats in ancient leather indicating their use in leather manufacturing. Such as the identification of plant lipids in Neolithic leathers from the contemporaneous Schnidejoch Pass site in Switzerland^7^.

The Iceman’s garments and quiver are from an assemblage of at least five different species of animal. The coat alone was a combination of at least four hides and two species: goat and sheep. This result may indicate a haphazard stitching together of clothing based upon materials that were available to the Iceman, as ancient rudimentary leather is posited to rapidly deteriorate after manufacture^6^,^12^. However, the leggings were composed of goat leather, which was also used in the manufacture of a 4,500-year-old leggings from Schnidejoch, Switzerland. This result lends support to the idea that Copper Age individuals in the Alpine region selected species for specific attributes when manufacturing clothing^8^. This may also indicate a functional choice of material based on flexibility or insulating potential.

Previous microscopic and proteomic analyses of the Iceman’s clothes could not resolve beyond genus taxonomy or surpass the technical challenges caused by absence of species specific markers^4^,^5^. Hollemeyer *et al*.^5^ noted that many domestic and wild species germane to the study were too closely related to distinguish PMFs (Peptide mass fingerprints) or that adequate reference databases did not exist. There are inconsistencies between the species identification of our study and Hollemeyer *et al*.^5^, contrary to our results they observed chamois and red deer PMFs in parts of the coat, Canid PMFs in the leggings, and cattle PMFs in the quiver. Different sampling locations on the same clothing may explain this disparity, as highlighted by our results from the coat analysis above.

The haplogroups identified in these samples from sheep, goat and cattle all fall within the domestic variability of the source animal in modern Europe, novel SNPs have been recovered that may have been lost from contemporary domestic haplogroups (Supplementary Table 3). In this study, the goat mitogenomes were identified as haplogroup A, yet a goat B1 haplogroup was observed in a study of 4,500-year-old swiss leggings^8^. The ancient distribution of domestic goat haplogroups are unknown, yet B1’s modern European distribution is restricted to Greece and Turkey^8^,^42^. Goat haplogroup B1 is more widespread in western Asia, but is closely related to haplogroup A. The Iceman and his leggings predate the Swiss find by c. 800 years, suggesting an overlap in the distribution of goat haplogroups that is no longer present in Europe. The sheep leather and fur were identified within haplogroup B1 from the three coat fragments and the loincloth. One sheep (coat 10304) mitogenome has the same distinct mutations at loci 16147 and 16440 reported by Olivieri *et al*.^13^ in a PCR analysis of fibres from a later excavation the Iceman’s find site. However, other mutations reported by Olivieri *et al*.^13^ were not present indicating that they identified sequences from yet another sheep used in the garment assemblage and recovered from the find site. The shoelace mitogenome closely resembled the cattle reference sequence of haplogroup T3, which is the predominant mitochondrial haplotype in European domestic cattle and is observed in high frequency throughout the Neolithic of Europe^43^.

The quiver was identified as roe deer, clustering near the contemporary central European clade. This is the first time this species has been identified as being part of the Iceman’s leather attire. Notably, red deer (*Cervus elaphus*) was identified from PCR of hair shafts at the finding site^14^ and contents of the Iceman’s stomach^44^, and from PMFs of the quiver^5^. The evidence that the Iceman utilised at least two deer species shows the variety of wild materials from which he subsisted. The hat sequence clusters inside the Western lineage of brown bears, suggesting regional continuity of this genetic marker. Previous studies of brown bears report complex phylogeography across its range, typified by overlapping ranges of divergent clades of brown bear and introgression with polar bears (*Ursus maritimus*) due to convoluted gene flow caused by Pleistocene climatic changes^40^,^45^. The assignment of the hat to brown bear is important given that previous studies could not resolve its taxonomy beyond *Carnivora*^5^, and secondly that it is unambiguously a wild species that was used for clothing manufacture.

Despite the Iceman being established as an agro-pastoralist^2^,^3^, the hat and quiver provide evidence of hunting and trapping of wild animals. Other studies have proposed parts of the diet, clothing and equipment as belonging to wild species such as wild canid (for the hat, species previously unresolved), red deer and chamois^5^,^14^. Rollo *et al*.^44^ demonstrated that the Iceman consumed red deer and alpine ibex (*Capra ibex*). The choices that Copper Age people made with respect to animals exploitation were likely dependent on availability, necessity, functionality and symbolism^46^. Given the diversity of the analysed material (each analysed fragment comes from a different animal), the lack of any obvious symbolism and the rapid degradation rate of ancient leather^11^ it would appear that the Iceman’s leathers and furs were first chosen haphazardly for subsistence as rudimentary leather pieces rapidly disintegrated and required replacement with strips of new hide. There is however likely to be a regional variation in the selection of wild and domestic populations throughout the Copper Age and the Iceman provides a window into the alpine region which may have been unlike other locations^46^.

These results are based on uni-parental maternal genetic markers, hence the authors cannot exclude the possibility that the analysed domestic species were sourced from herds that were re-stocked with wild males as occurred between cattle and aurochsen populations as is postulated in ancient Britain^31^. Whole genome sequencing of the Iceman’s attire in the future may increase our knowledge to the degree of introgression with local populations or breeding selection for functional genes.

## Materials and methods

### Ancient DNA extraction and library preparation

DNA extractions were based on the protocol of Gamba *et al*.^47^ and conducted in a dedicated ancient DNA laboratory in Trinity College Dublin. Barcoded Illumina NGS libraries were prepared following the protocol of Meyer and Kircher^48^ with modifications by Gamba *et al*.^47^. The one exception was the hat, which was extracted and library prepared at the clean room facility at the EURAC - Institute for Mummies and the Iceman, Bolzano. The extraction protocol for the hat was adapted from Rohland *et al*.^49^. The hat library was subsequently indexed and captured at Trinity College Dublin.

### Mitochondrial DNA enrichment and Sequencing

Eight DNA extracts from five articles of the Iceman’s clothing and one from the quiver were selected for mtDNA enrichment. Firstly, libraries were shotgun sequenced (excluding the hat) on a Illumina MiSeq to check the quality of the extracted DNA and the percentage endogenous content. Prior to sequencing, the indexed libraries were pooled and enriched for mitochondrial genomes using a custom RNA capture produced from reference domesticate mtDNA genomes by MYcorarray (Mycroarray, 5692 Plymouth Road, Ann Arbor, MI 48105) following the manufacturer’s instructions. The shotgun and captured libraries were both sequenced on the Illumina MiSeq platform at TrinSeq St James’s Hospital Dublin.

### Processing of raw data and mapping

Universal Illumina adapters were trimmed from the 3′ end of raw sequences using Clip&Merge^50^ with a minimum read length of 30 base pairs (bp) and 2 bp clipped from both the 5′ and 3′ end termini. Fastq Screen (http://www.bioinformatics.babraham.ac.uk/projects/fastq_screen) was used as a first-pass species identification using a cohort of reference animal mitogenomes (cattle, sheep, goat, deer, bear, human, ibex, pig, chamois and dog) obtained from the refseq database at NCBI^51^ (Supplementary Table 4). Fastq Screen displays which reference the sequenced libraries preferentially map to and have the most species specific reads. Reads were then re-mapped to the most similar reference mitogenome with BWA^52^ using standard parameters for aDNA^53^. For each mitochondrial reference (Supplementary Table 1), the first 30bp were manually copied and pasted to the end of the reference to compensate for the circular nature of the mitochondrial genome^54^. PCR duplicate (clonal) reads were removed using SAMtools^55^ and ancient DNA authenticity was assessed using mapDamage^32^ (analysing adapter removed but unclipped data). MapDamage was further used to rescale the base quality of putatively damaged bases i.e. G to A or C to T transitions approaching the 5′ and 3 termini. With one exception for sheep whose mtDNA genome contains a repeat expansion^38^, all reads were filtered for a minimum mapping quality of 30 with the SAMtools view^52^. Rough mitochondrial cross-contamination estimates from other animals were made following the methods adapted from Sánchez-Quinto *et al*.^56^ and Teasdale *et al*.^20^ analysing mismatches at haplogroup defining positions.

### Phylogenetic analysis and haplogroup assignment of samples

A consensus fasta sequence was generated for each sample using ANGSD (analaysis of next generation sequencing data) tool^57^, filtering out base quality below 30 and making a majority consensus call from overlapping reads. Bam files were visualised with IGV^35^ during construction of fasta consensus sequences to investigate if any polymorphisms in the consensus were confounded by low coverage (less than 3 times covered) and to identify INDELs that may have been overlooked during consensus calling.

Reference mitochondrial sequences were acquired from the NCBI database, where possible using a RefSeq sequence. The sheep reference sequence AF010406, used by Dometree^34^, corresponds exactly to the sheep reference NC_001941 from Hiendleder *et al*.^58^. The consensus sequences were aligned to a cohort of mitogenomes from NCBI (Supplementary Table 1) using MUSCLE^59^ with maximum of two iterations, which represent modern diversity in each species and appropriate outgroups to root the phylogenetic trees, and where possible ancient data sequenced for that species (Supplementary Table 1). Neighbour-joining (NJ), maximum-likelihood (ML), and maximum-parsimony (MP) phylogenetic trees were constructed in Seaview^60^. The Jukes-Cantor substitution model, 100 bootstrap iterations and gaps ignored setting were used for neighbour-joining. The generalised time reversible (GTR) and 100 bootstraps settings were used for the maximum-likelihood trees. The MP used a less thorough tree search and 100 bootstraps. Ulitmately, the ML trees were chosen and visualised using FigTree (http://tree.bio.ed.ac.uk/software/figtree/). However, bootstrap values from NJ and MP above 90 were considered. Loci were excluded from the phylogenetic trees if they were cited in previous publications as confounding analysis or caused polyphylogeny among established haplogroups^34^,^36^,^38^,^39^,^40^,^54^.

Phylogenetic analysis for all species was based on an assemblage of mitogenomes within their respective genera. For sheep (*Ovis aries*) this was based on an assemblage of *Ovis* mitogenomes from Meadows *et al*.^38^, Dometree^34^ and Lancioni *et al*.^61^, using the haplgroup nomenclature of the later. For cattle (*Bos taurus*) phylogeny was based on Dometree^34^, Bonfiglio *et al*.^62^, Zhang *et al*.^54^ and Achilli *et al*.^37^. Goat (*Capra hircus aegagrus*) phylogenies were based on Dometree^34^, Colli *et al*.^42^ and Hassanin *et al*.^36^. Mitogenomes identified as clustering within the major haplogroups of cattle, sheep and goat by Peng *et al*.^34^ were added to the phylogenies to increase the resolution. Some of the nomenclatures for the sheep and goat subhaplogroups were updated from the dometree website using the above publications. Haplogroups and novel haplotypes for domestic species were identified by analysing the consensus sequences with MitoToolPy^34^ tool against a dataset of established haplotypes for domestic animals^34^. In this study, haplogroup assignments incorporated the above publications for the inclusion of up-to-date haplotypes. Roe deer (*Capreolus capreolus)* sequence data were aligned against and assemblage of Eurasian Capreolus variety^39^. Brown bear (*Ursus arctos*) sequence data were aligned with an assemblage of European, Polar, Eurasian and North American variety^40^,^45^.

For sequenced libraries with insufficient coverage to fully reconstruct mitogenomes, the partial data was tentatively converted to a consensus sequence with gap sites. The sequences were then inspected using IGV (the integrative genome visualizer)^35^. The data were aligned using methods as above, in addition GBlocks^63^ was used (except for Sample OCL2, which included gaps) to create a subset of aligned data that excluded all gaps from phylogenetic analysis.

## Additional Information

**Accession codes**: Sequence reads were uploaded to the Sequence Read Archive at the ENA (European Nucleotide Archive), study accession PRJEB13144.

**How to cite this article**: O’Sullivan, N. J. *et al*. A whole mitochondria analysis of the Tyrolean Iceman’s leather provides insights into the animal sources of Copper Age clothing. *Sci. Rep.* **6**, 31279; doi: 10.1038/srep31279 (2016).

## Supplementary Material

Supplementary Information

## Figures and Tables

**Table 1:** Summary statistics and identification of sequenced libraries of this study.

Sample	Species^a^	Haplo- groups^b^	Reads shotgun (#)	Mapped unique reads shotgun (#)	Reads capture (#)	Mapped unique reads capture (#)	Enrich-ment fold^c^	Coverage (X)	Conta- mination (%)
Coat (10304)	Sheep	B1	824,279	14	1,739,134	7,028	238	20.6	0.43
Dark coat (OCD)	Sheep	B1	723,348	6	2,422,032	5,141	256	15.5	1.32
Light coat (OCL2)*	Sheep	B1	533,933	5	594,102	1,197	215	3.6	3.97
Loincloth (OLC)	Sheep	B1	587,776	32	1,287,892	14,879	212	48.3	1.58
Coat (O314)	Goat	A	870,940	2,763	1,993,094	29,521	5	104.6	0.38
Leggings (OL)	Goat	A	962,864	102	4,900,933	30,502	59	110	0.54
Shoelace (OSA)	Cattle	T3	13,234,516	2,169	72,794	7,398	620	22.4	4.76
Quiver (OCA)*	Roe Deer	Central European	4,947,201	194	364,953	822	57	2.0	NA
Hat (OH)*	Brown bear	Western Lineage	NA	NA	21,672	151	NA	0.4	NA

^*^Indicates partially reconstructed mitogenomes.

^a^Fastq screen species identification.

^b^The haplogroups were identified for each domestic species using the MitoToolPy tool and the observation of previously defined haplotypes for domesticated species. Wild species clades were assigned based on how they clustered in their respective phylogenies (Fig. 2).

^c^Enrichment fold roughly estimates the success of targeted enrichment. It is based on a downsampling of shotgun and capture reads to a comparable number. The downsampled number of captured reads is divided by the number of shotgun reads. Two exceptions are for sample OCA and OSA in which the shotgun libraries were more abundant and hence were downsampled rather than the capture libraries.
