# Supplementary material for: A whole mitochondria analysis of the Tyrolean Iceman’s leather provides insights into the animal sources of Copper Age clothing
Source: Sci Rep. 2016 Aug 18;6:31279. doi: 10.1038/srep31279 (PMC4989873; doi:10.1038/srep31279)
Supplement: Supplementary Information [file srep31279-s1.pdf]

Supplementary document: A whole mitochondria analysis of the Tyrolean Iceman's leather provides insights into the animal sources of copper age clothing

Author list and affiliations

*Niall J. O'Sullivan[1, 3]

Matthew D. Teasdale[2]

Valeria Mattiangeli[2]

Frank Maixner[1]

Ron Pinhasi[3]

Daniel G. Bradley[2]

Albert Zink[1]

[1]Institute for Mummies and the Iceman, EURAC research, 39100 Bolzano, Italy

[2]Smurfit Institute of Genetics, University of Dublin, Trinity College, Dublin 2, Ireland

[3]School of Archaeology, University College Dublin, Belfield, Dublin 4, Ireland

**Species Phylogeny (reference accession code)**	**Accession codes of Mitogenomes and associated haplogroup**	**Outgroups (accession codes) and species**
**Sheep (*Ovis aries*) (AF010406)**	KF302447_B, KF302448_B1, AF010406_B1a1b_reference, EF490451_B1a_ 7G65F4, EF490454_B1a2_Oe173, EF490455_B1a_1LL2, EF490456_B1a_OLL15, HM236174_A1b_kk1, HM236175_A1b, HM236176_B1a4, HM236178_C_kk2, HM236179_C_mk4, HM236181_D, HM236182_E, HM236183_E, HM236185_B1a3_mouflon, KF302440_A1, KF302445_A1, KF302449_B1a1a, KF302450_B1a1a, KF302452_B1a2a, KF302453_B1a2a, KF302455_B1a2a, KF302457_B1a2a1a, KF302458_B1a2a1a, KF302459_B1a2a1a, KF302460_B1a2b, KF302461_B1a2b, KF302462_B1a2b, KF977845_B1a2_ Djallonke, KF977846_B1a1b_ Sahelian	HM236187_Ovis_vignei, HM236188_Ovis_ammon ,HM236189_Ovis_vignei
**Goat (*Capra aegagrus hircus*) (NC_005044. 2)**	GU068049_A3, GU295658_B_isolate V07-146, KF952601_B, KJ192209_A2a, KJ192210_A2a1, KJ192211_A2a1, KJ192212_A2b2a, KJ192213_A2b2a, KJ192214_A2c1b, KJ192215_A2c1b, KJ192216_A1, KJ192217_A1b1a, KJ192218_A1a1a, KJ192219_A1a1a, KJ192220_A2b1a, KJ192221_A2b1a, KJ192222_A2b2b, KJ192223_A2b2b, KJ192224_A2c1a, KJ192225_A2c1a, KJ192226_A1a1, KJ192227_A1a, KJ192228_A2c, KJ192229_A2c, KJ192230_A1b2, KJ192231_A1b2, KJ192232_A2b1, KJ192234_A1b1a,	FJ207525_*Capra falconeri,* FJ207526_*Capra ibex*

	KJ192235_A1b1, KJ192236_C	
**Cattle (*Bos taurus*) (V00654)**	AY126697_I1, AY676861_T3d1, AY676862_T3r, AY676864_T3g, AY676873_T3o, DQ124375_T4a, DQ124376_T3k1, DQ124377_T4, DQ124391_T3n1, DQ124409_T3c, DQ124416_T3b, DQ124418_T3a1a, EU177824_T3p, EU177826_T3q1, EU177829_T3j, EU177852_T2d, EU177853_T2, EU177857_T2b, EU177860_T2c, EU177865_T5b, EU177868_I1, FJ971084_R1a, FJ971085_R1a, GU947012_T3f1, HQ184030_Q2, HQ184036_Q1, JN817304_T1d1, JN817309_T1c1a1, JN817313_T1a, JN817330_T1d1, JN817335_T1a1, JN817343_T1f, JN817348_T1b, JN817351_T1b1a, JQ437479_primigenius, GU985279_CPC98, JQ967333_T3s, KC153973_T1e, KF163072_T1c, KF163081_T1d, KF525852_C(Ancient Chinese Cattle)	EF494178.1_*Bos grunniens*, GU947006.1_*Bison bison*, JN632602.1_*Bison bonasus*
**Brown Bear (*Ursus arctos*) (HQ685964)**	AF303110_Eastern, AF303111_Ursus_maritimus, AJ428577.1_Greenland, EU497665.1_Western, GU573486_isolate_12WH,GU573487_isolate_A91-05,GU573488_Ursus_maritimus_,GU573489_isolate_14KB_,GU573490_Ursus_maritimus_isolate_495, GU573491_isolate_76824,HQ685901_isolate_1,HQ685903.1_isolate_3, HQ685929_isolate_29,HQ685942_isolate_42, HQ685957_isolate_57,HQ685960_isolate_60, JX196367_isolate_KEN10-UAR100,JX196369_isolate_ABC1-051711,AP012559_isolate_834	*Ursus spelaeus* (EU327344), *Ursus americanus* (NC_003426) and *Ursus thibetanus* (NC_011118)
**Roe Deer (*Capreolus capreolus*)**	KJ681480-KJ681491	*Capreolus pygargus* (KJ681491- KJ681495), *Alces alces* (NC_020677)

**(NC_020684)**		and *Hydropotes inermis* (NC_011821)

Supplementary Table 1 | Accession codes for mitochondrial genomes downloaded from NCBI for phylogenetic tree construction.

SAMPLE	TOTAL SHOTGUN READS	SHOTGUN READS AFTER CLIPPING	SHOTGUN TOTAL MAPPED READS	FILTERED UNIQUE MAPPED READS	SHOTGUN MT COVERAGE (X)	TOTAL CAPTURED READS	CAPTURE READS AFTER CLIPPING	CAPTURE TOTAL MAPPED READS	FILTERED UNIQUE MAPPED READS	CAPTURE MT COVERAGE (X)	CAPTURE AVERAGE READ LENGTH
**10304**	824279	790340	15	14	0.04	1739134	1588194	38293	7028	20.6	55.7
**O314**	870940	832339	3042	2763	7.99	1993094	1891808	1404087	29801	104.6	63
**OCA**	4947201	4734128	213	194	0.41	364953	313309	1512	822	2.0	53
**OCD**	723348	691219	6	6	0.02	2422032	2295615	23580	5141	15.5	65
**OCL2**	533933	508963	5	5	0.01	594102	562100	6420	1,197	3.6	65
**OLC**	587776	561880	32	32	0.08	1287892	1225548	134420	14879	48.3	59
**OL**	962864	913673	103	102	0.34	4900933	4635327	847450	30782	110	64
**OSA**	13234516	12664087	2333	2169	5.92	72794	66300	16072	7398	22.4	54
**OH**	NA	NA	NA	NA	NA	21672	6960	1627	151	0.4	57

Supplementary table 2 | Sequencing output for shotgun and capture libraries.

Sample	Species	Haplogroups	Polymorphisms
**OSA**	Cattle	T3	**169G,215+T,587+C,2536A,9682C,13310C,** 1599deletion
**O314**	Goat	A	**203C,1849T,2207C,2849C,3194G, 3468C,4032C,5601T,6096C, 7052A,7342A, 7660C,7839C,7885T,8055G, 8078T,8480C,8635C,8703T,8730C,8818C,8959T,9010T, 9826T,10479G,10638T,10686C,10813C, 11364C,11691G,11702A,11872G, 12383T,13100T,13178C,13280G,13286C,13681A, 13741C,13756T,13964A,14461T,14746C,14795A, 15004C,15220T,** 1119deletion, 7027T,15543C,15631A,15637T,15728G,15747A,15807T, 15811C,15843G,15893C,15913G,16045C,16083T,16232C,16435A,16440C
**OL**	Goat	A	**203C,1849T,2207C,2849C,3194G, 3468C,4032C,5601T,6096C,7052A,7342A,7660C,7839C, 7885T, 8055G,8078T,8480C, 8635C,8703T,8730C,8818C,8959T, 9010T, 9826T,10479G,10638T,10686C,10813C, 11364C, 11691G,11702A,11872G,12383T, 13100T,13178C,13280G, 13286C,13681A,13741C,13756T, 13964A,14461T,14746C,14795A,15004C,15220T,** 1119deletion, 3222A,11558T, 12140A, 15462T,15543C,15631A,15637T,15728G,15747A,15807T,15811C,15843G,15893C,15913G,16045C,16083T,16232C,16458T,16485C,16504deletion,16515G
**10304**	Sheep	B1	**281C,566+G,1729+C,3543A,6615A,7500A,8264C,8651T,9375G,11668A,11710deletion,12539C,12571C,13199G, 13813C,14055C,15783T,15820T,16128T,16342+C,16343C,16472deletion,** 3092T,3230C,7983C,12494T,13375G,15579A,15923G,16147C,16440C,16466C
**OCD**	Sheep	B1	**281C,566+G,1729+C,3543A,6615A,7500A,8264C,8651T,9375G,11710deletion,11273T,11473G,11668A,12539C, 12571C,13199G,13813C,14055C,14653A,15721C,15783T,15800T,15820T,16128T,16342+C,16343C,163472deletion,** 6965T,6975T,14871C,15923G,16036A,16392C
**OCL2***	Sheep	B1	**281C,566+G,3543A,6615A,7500A,8264C, 8651T, 9375G,11668A,11710deletion,12539C,12571C,13199G, 14055C,15783T,16128T,16342+C,16343C,163472deletion,** 1849A,2141A,2144G,2148T,2443T,2666T,5350C,7983C, 8927G,10615T,13549T,14530G,16392C
**OLC**	Sheep	B1	**281C,566+G,1729+C,3543A,6615A,7500A,8264C,8651T,9375G,11668A,11710deletion,12539C,12571C,13199G, 13813C,14055C,15800T,15820T,16128T,16342+C,16343C, 16472deletion,** 2801C,11273T,11473G,14653A,14871C,15923G,16036A,16392C

Supplementary table 3 | Identified haplogroups for captured libraries made from consensus mapped reads. Polymorphisms listed in bold are expected haplotypes for each haplogroup, polymorphisms in regular font are mutations not yet associated with identified haplogroups. All polymorphisms are relative to the mapped reference mitogenome, NCBI accession codes are in Supplementary table 4. *OCL2 is a partial reconstruction with low average coverage, missing sites include: 1-8np, 405-418np, 1678-1800np, 2216-2316np, 2855-

2930np, 3471-3514np, 5527-5556np, 5604-5639np, 6393-6415np, 6760-6784np, 7515-7541np, 7611-7644np, 8735-8794np, 9029-9067np, 11604-11634np, 12420-12466np, 12723-12767np, 13288-13362np, 16247-16289np.

Species	Accession code
**Brown bear (*Ursus arctos*)**	HQ685964
**Chamois (*Rupicapra rupicapra)***	NC_020633
**Cattle (*Bos taurus*)**	V00654
**Roe deer (*Capreolus capreolus*)**	NC_020684
**Goat (*Capra aegagrus hircus*)**	NC_005044.2
**Human (*Homo sapien*)**	NC_012920
**Ibex (*Capra ibex*)**	NC_020623
**Pig (*Sus scrofa*)**	NC_012095
**Sheep (*Ovis aries*)**	AF010406

Supplementary table 4 | Reference mitogenomes used for species assignment. Used default parameters of Fastq_screen (http://www.bioinformatics.babraham.ac.uk/projects/fastq_screen/) to find which references raw sequenced reads preferentially align.

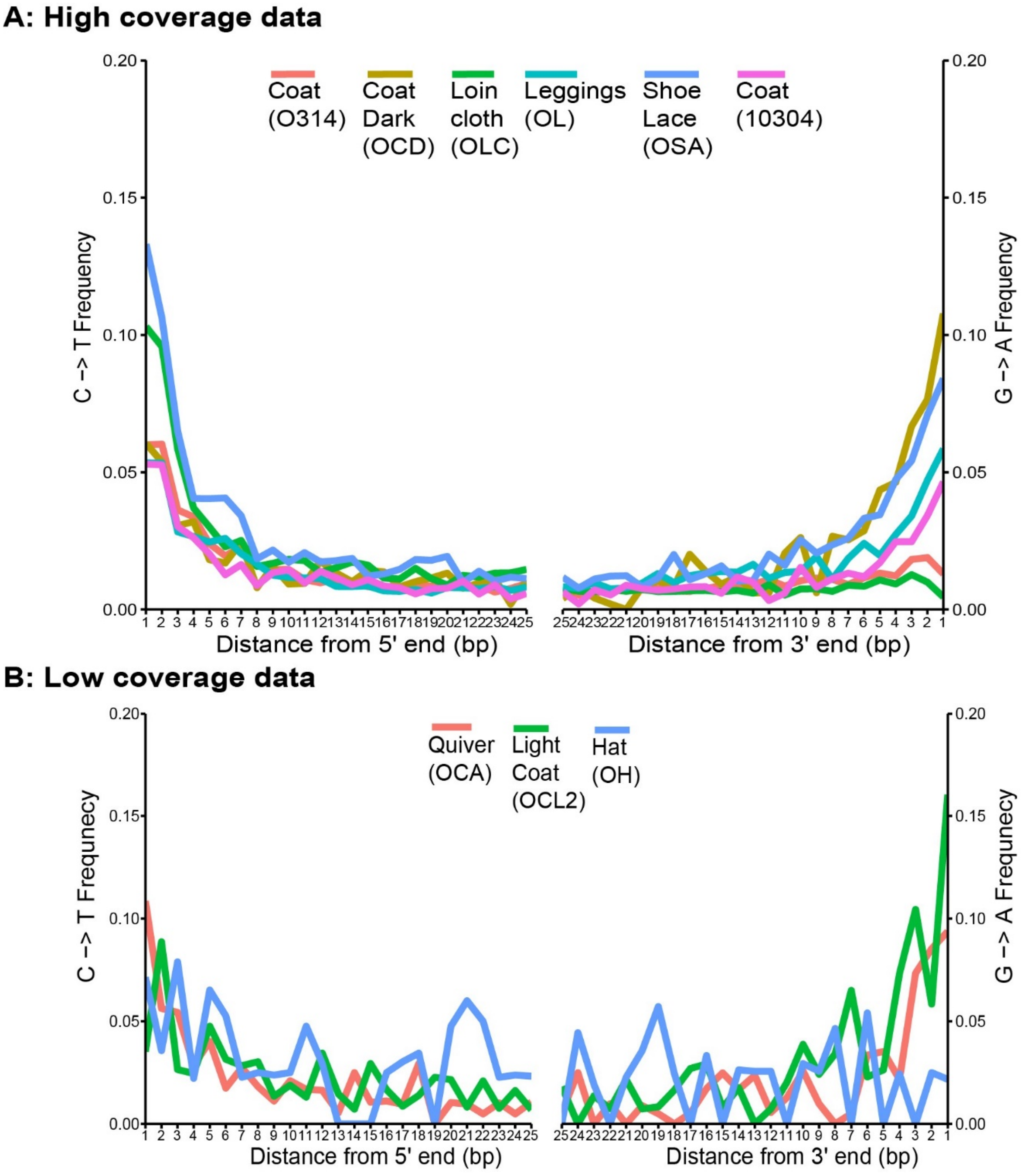

Supplementary Figure 1 | mapDamage deamination plot. (**a**) Six high coverage enriched mitogenome libraries. (**b**) Three low coverage enriched mitogenome libraries.

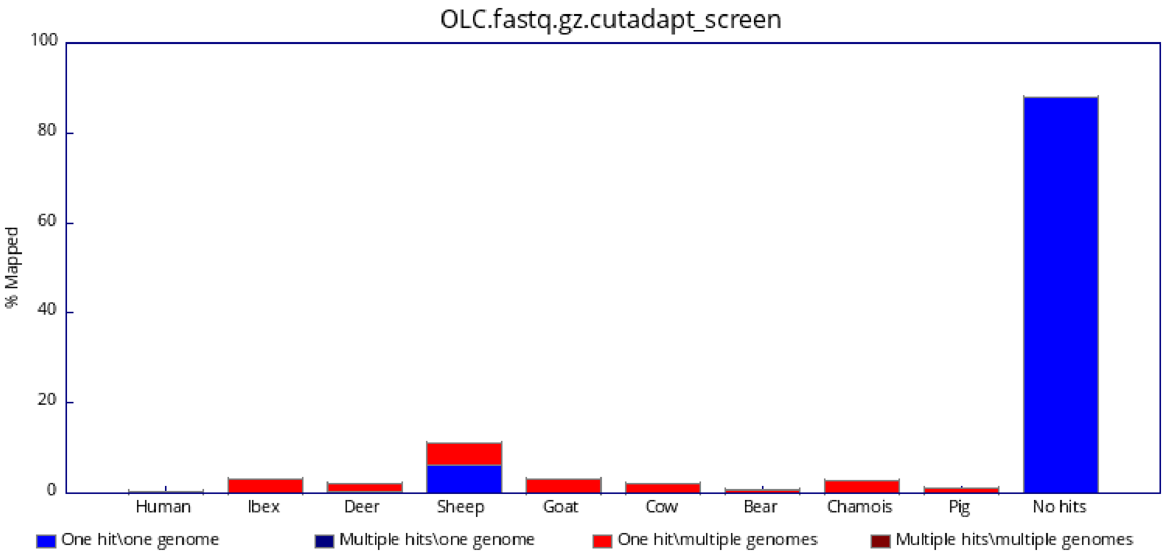

Supplementary Figure 2 | Fastq_screen. Initial species assignment for Loincloth from enriched library.

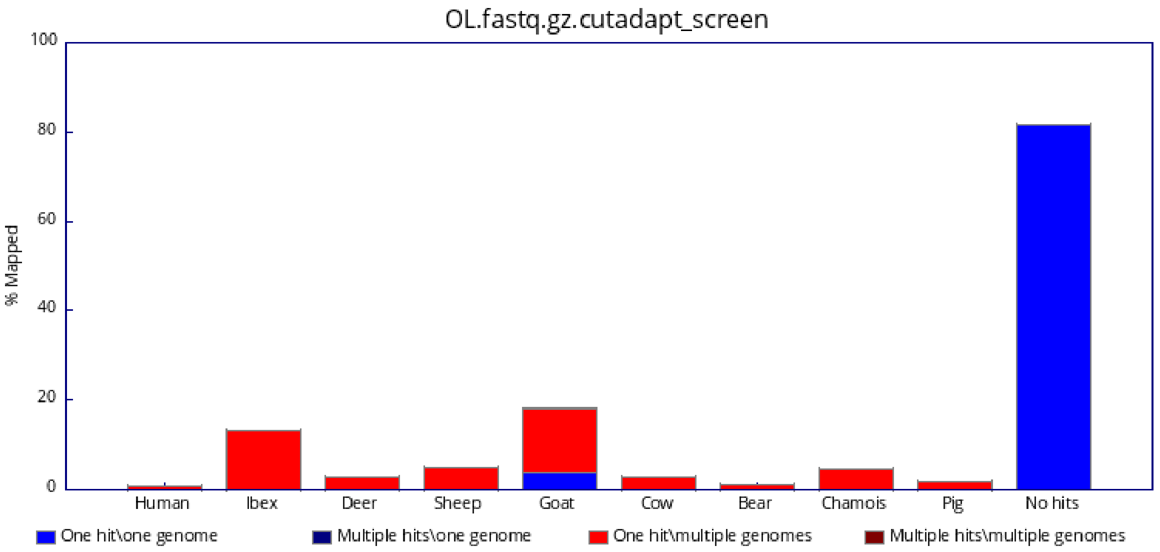

Supplementary Figure 3 | Fastq_screen. Initial species assignment for leggings from the enriched library.

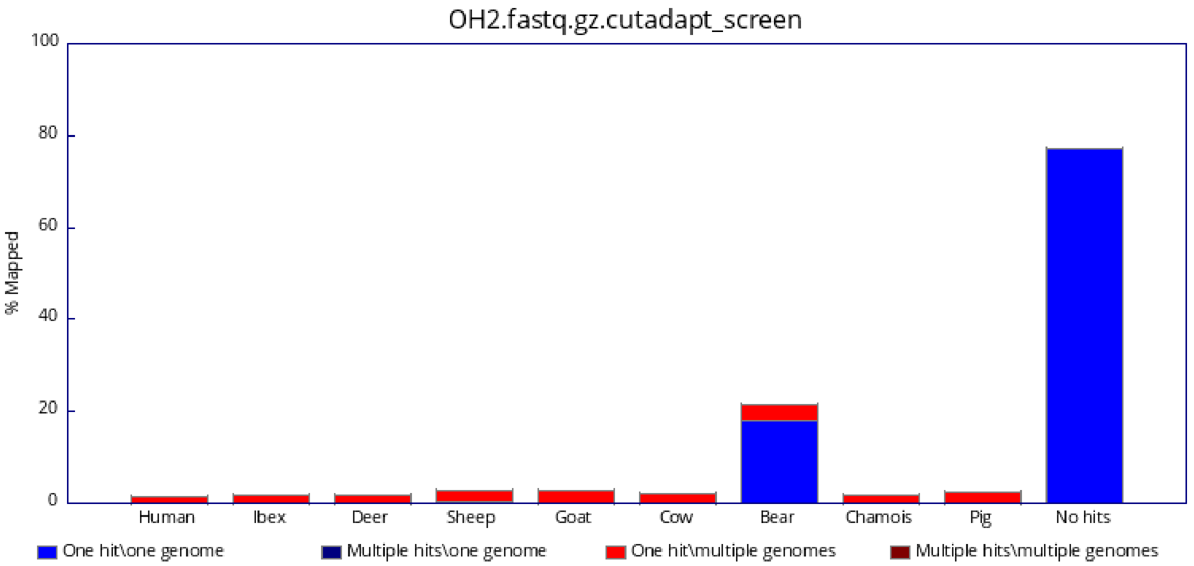

Supplementary Figure 4 | Fastq_screen. Initial species assignment for the hat from enriched library.

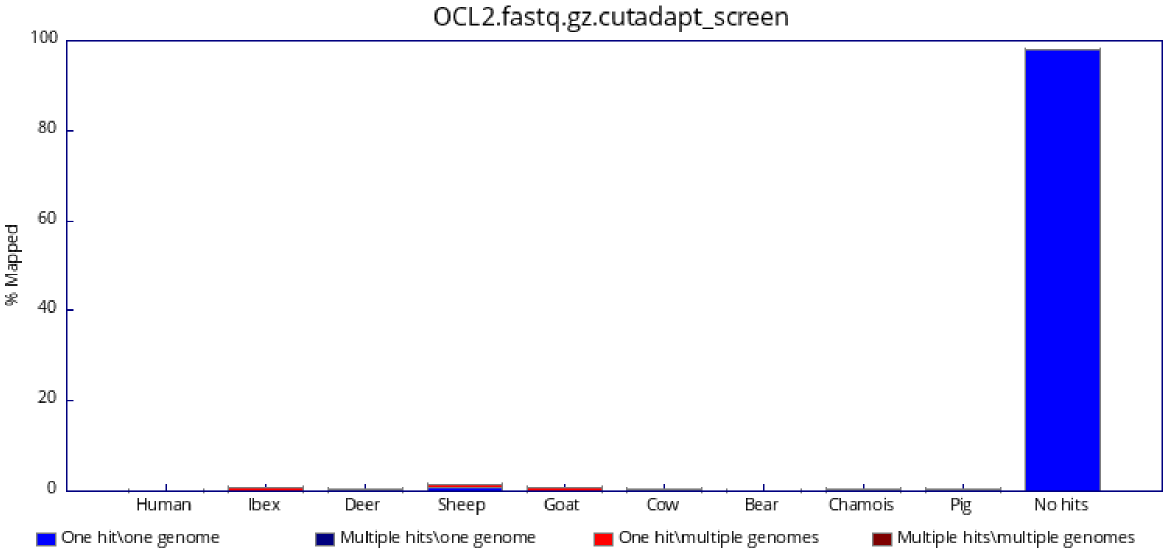

Fuu

Supplementary Figure 5 | Fastq_screen. Initial species assignment for the light coat from enriched library.

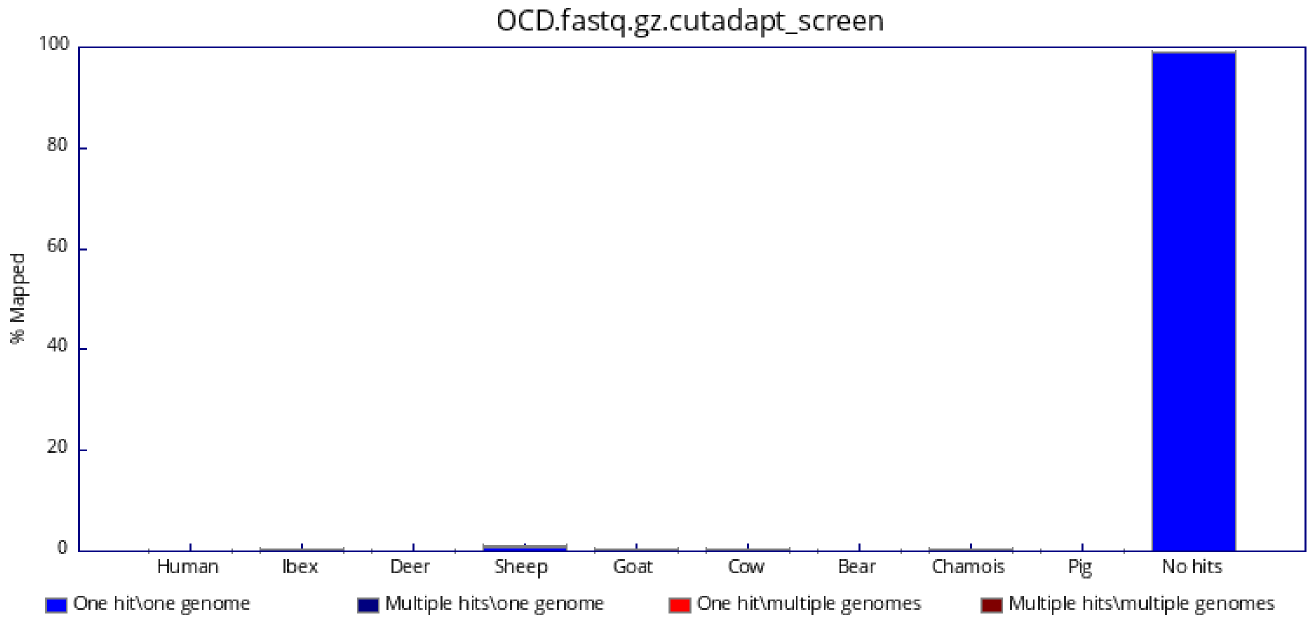

Supplementary Figure 6 | Fastq_screen. Initial species assignemnt for the dark coat from the enriched library.

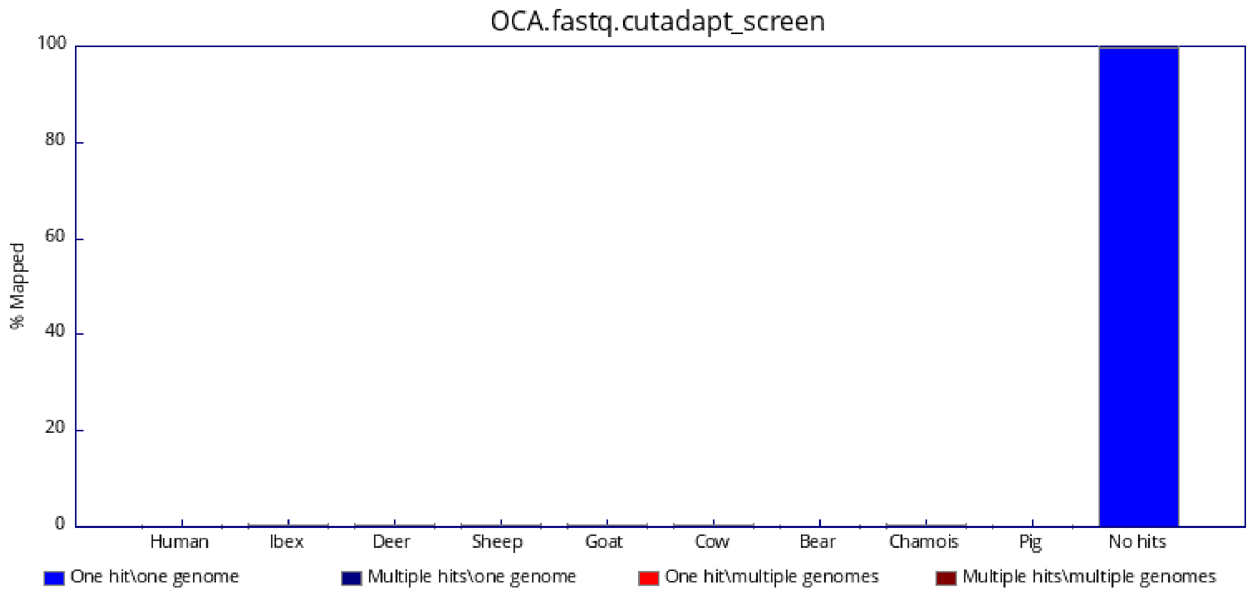

Supplementary Figure 7 | Fastq_screen. Initial species assignment for the quiver from enriched library.

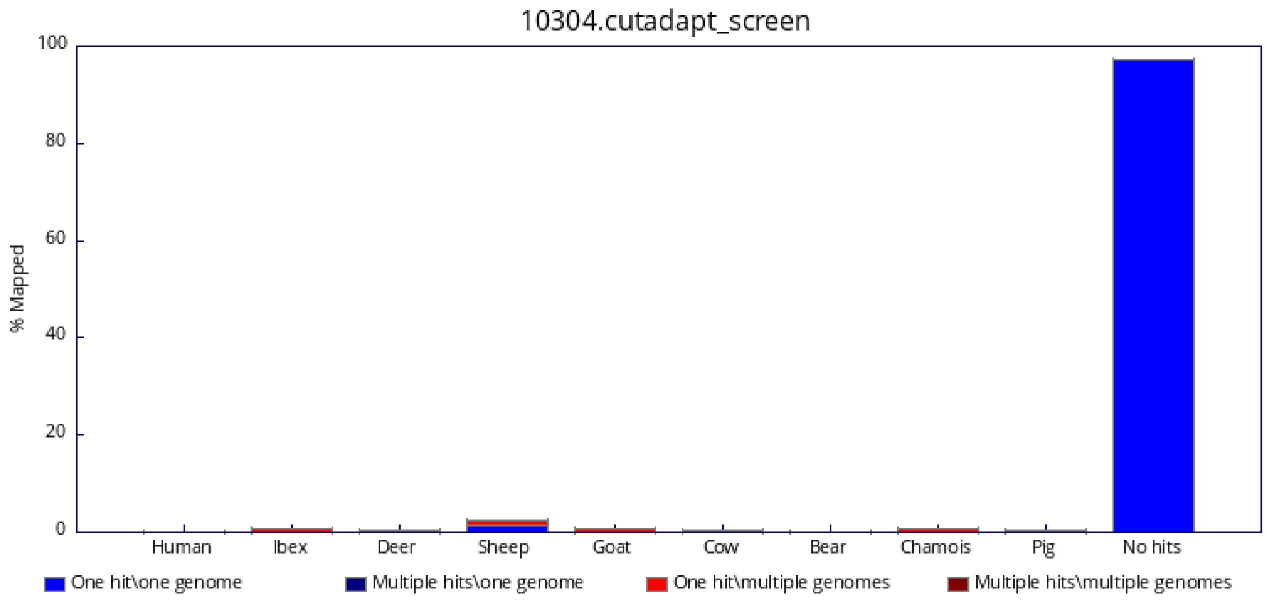

Supplementary Figure 8 | Fastq_screen. Initial species assignment for coat sample 10304 from the enriched library.

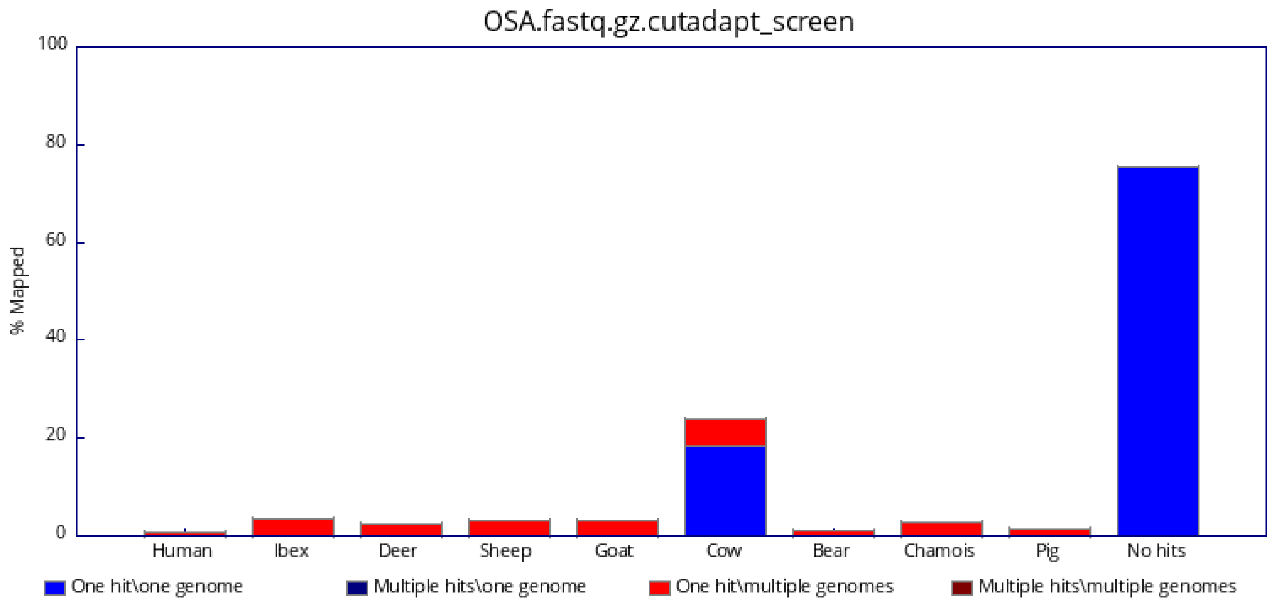

Supplementary Figure 9 | Fastq_screen. Initial species assignment for the shoelace sample from the enriched library.

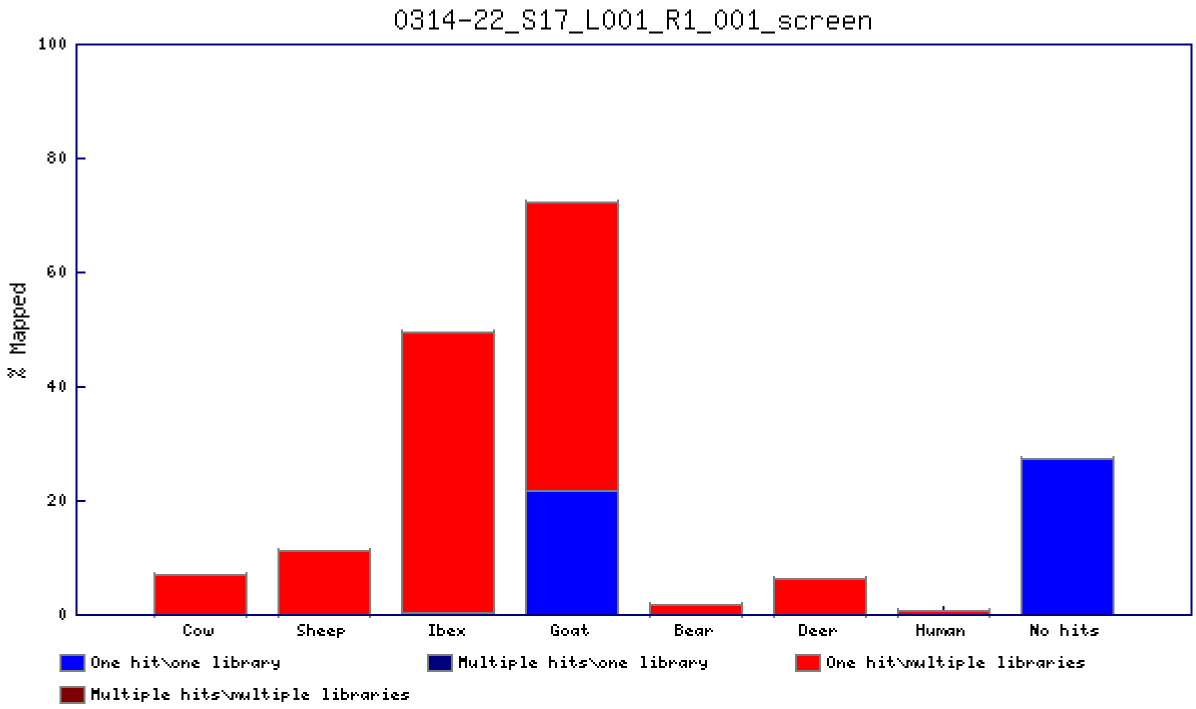

Supplementary Figure 10 | Fastq_screen. Initial species assignment for the coat sample 0314 from the enriched library.

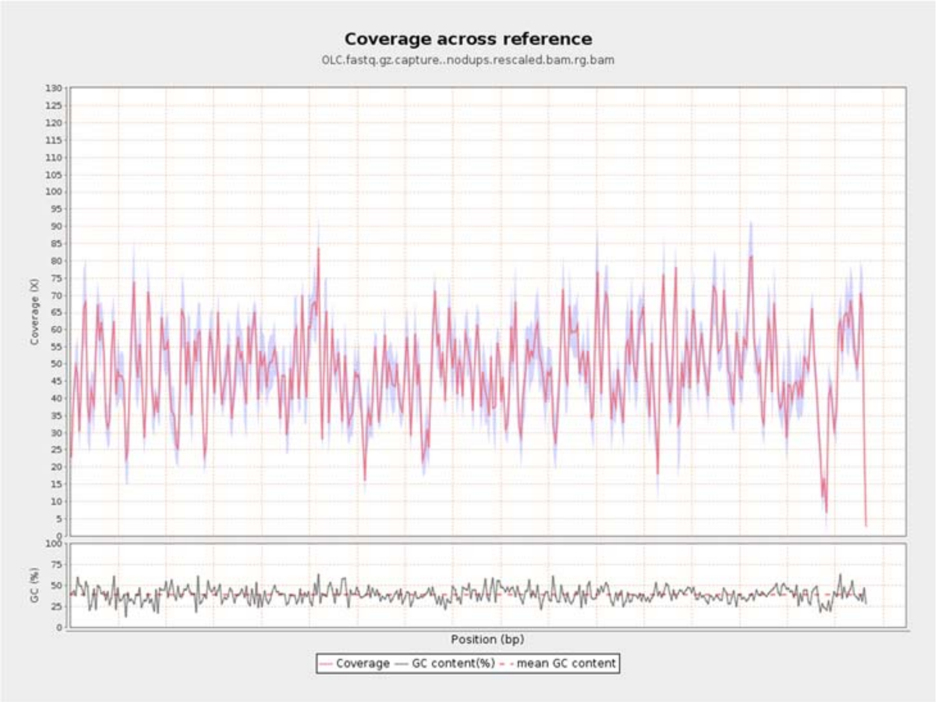

Supplementary Figure 11 | Coverage across the reference. Captured loincloth library (OLC).

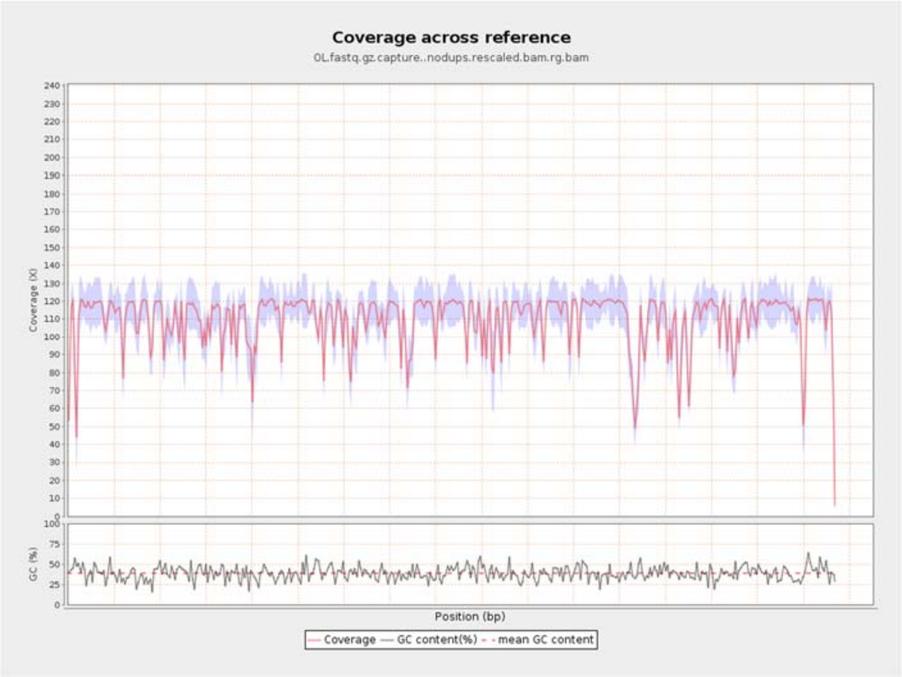

Supplementary Figure 12 | Coverage across the reference. Captured leggings library (OL).

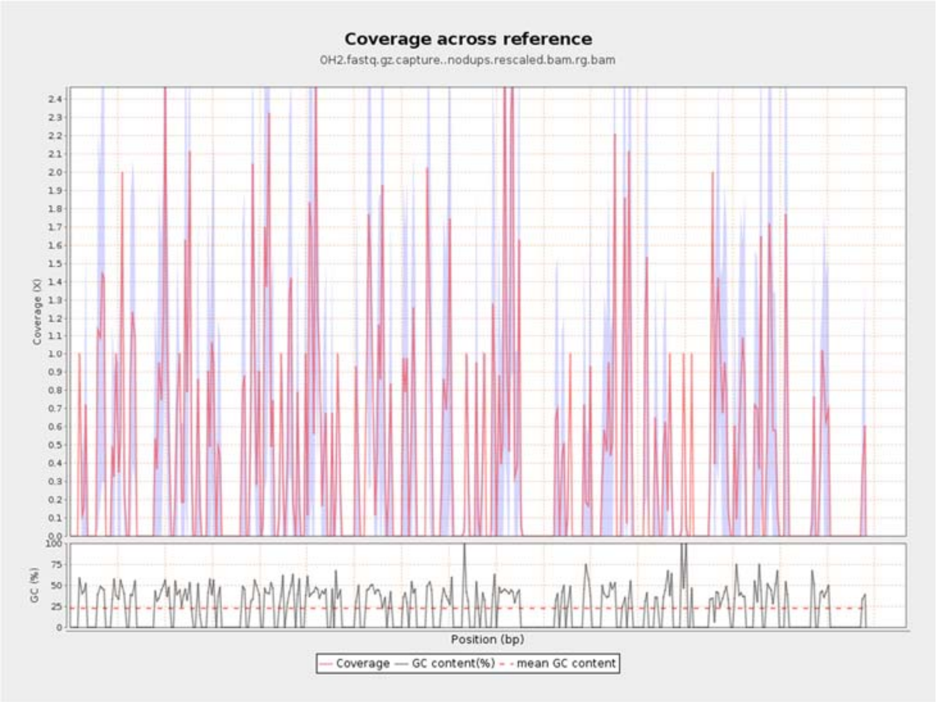

Supplementary Figure 13 | Coverage across the reference. Captured hat library (OH).

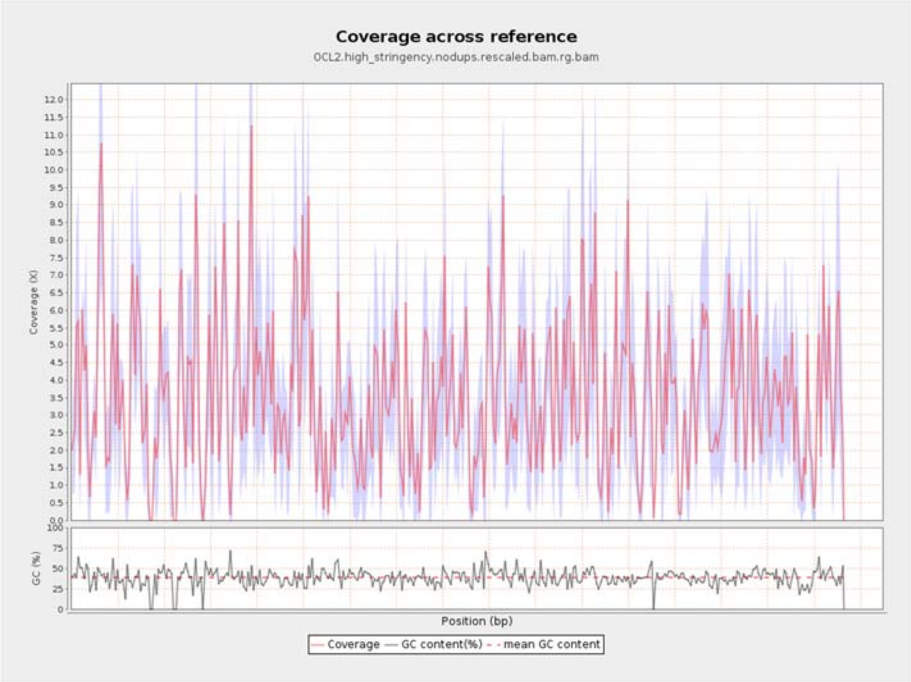

Supplementary Figure 14 | Coverage across the reference. Captured light coat library (OCL2).

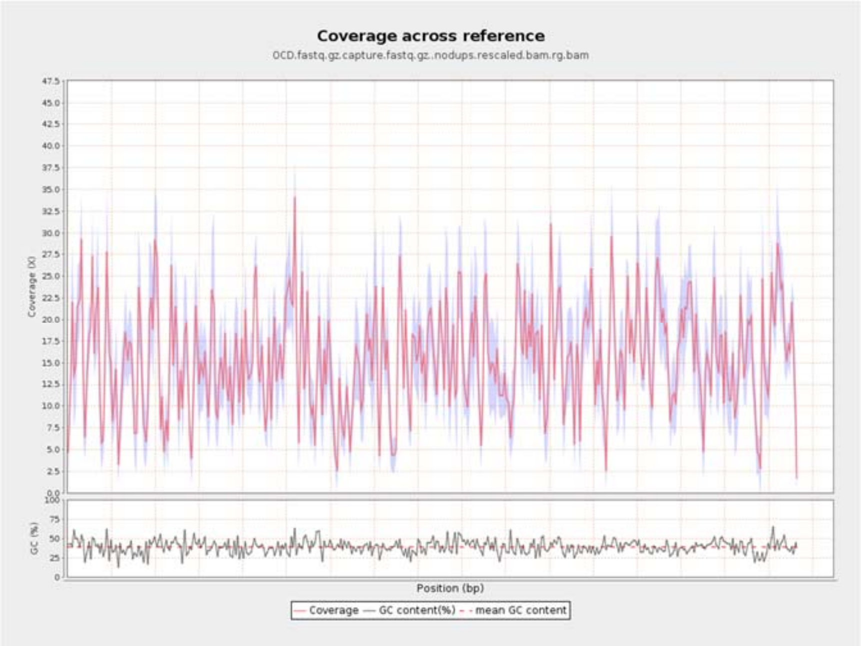

Supplementary Figure 15 | Coverage across the reference. Captured dark coat library (OCD).

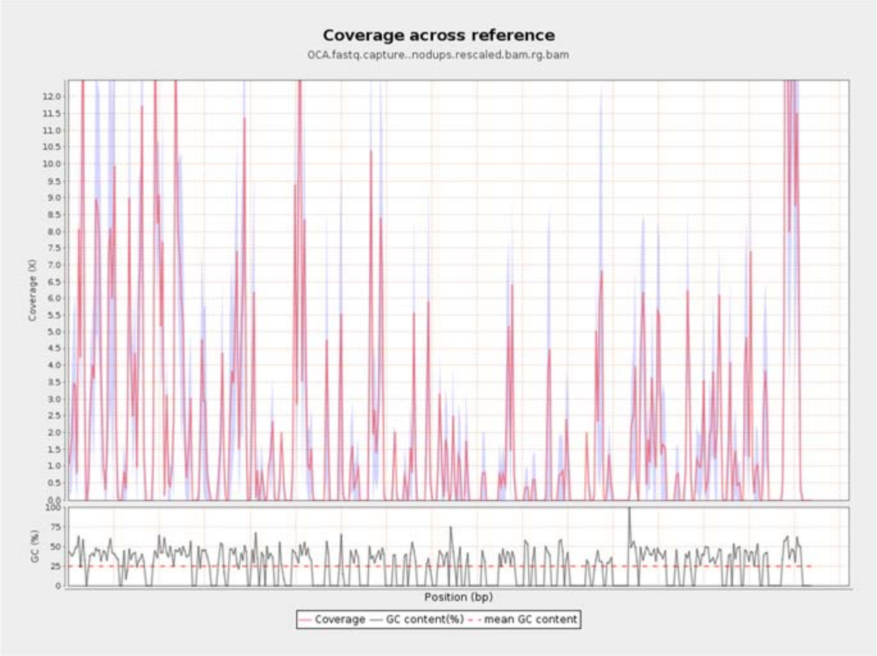

Supplementary Figure 16 | Coverage across the reference. Captured quiver library (OCA).

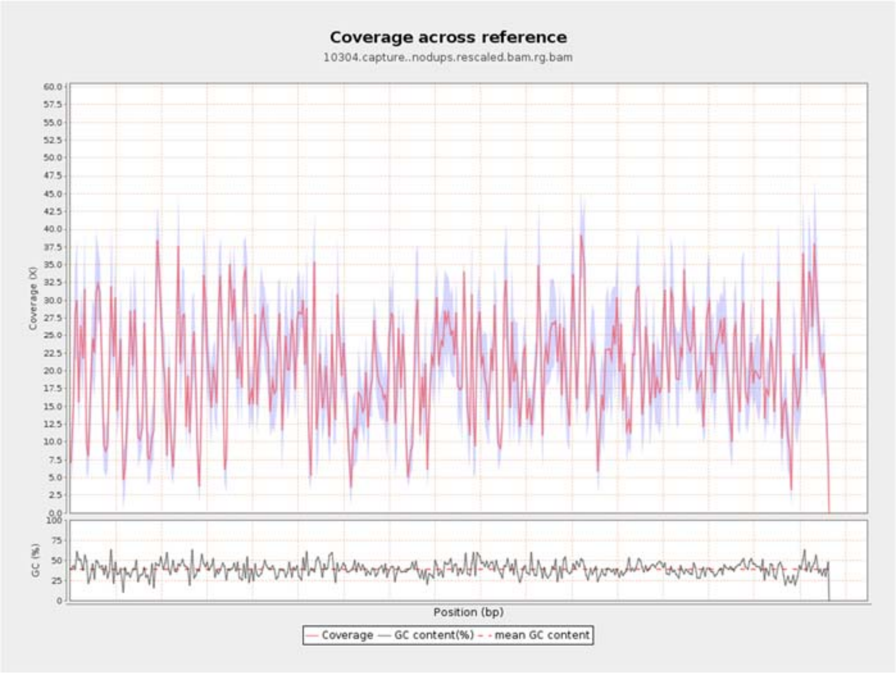

Supplementary Figure 17 | Coverage across the reference. Captured coat library (10304).

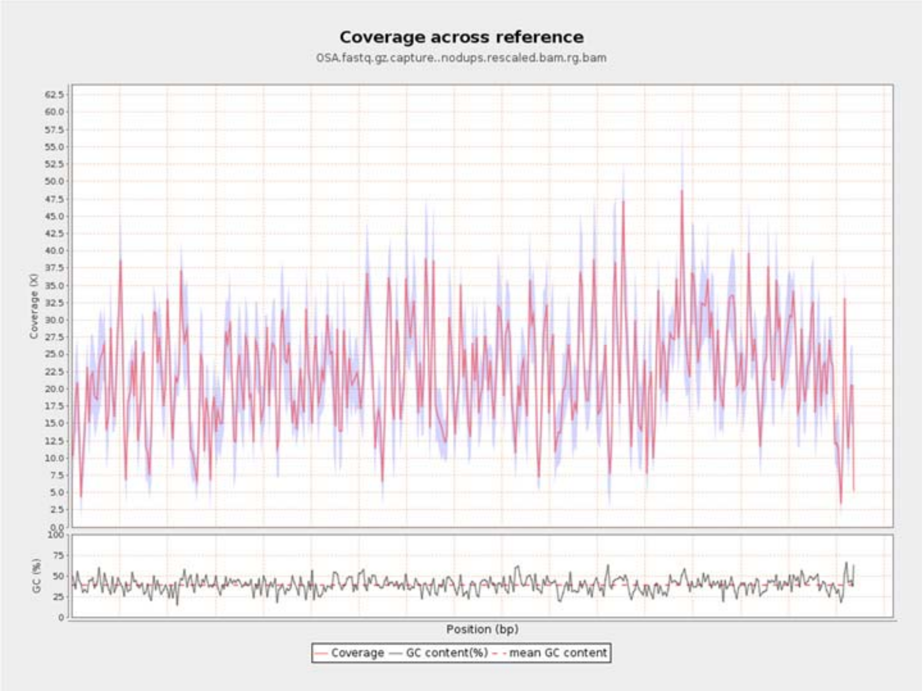

Supplementary Figure 18 | Coverage across the reference. Captured shoe library (OSA).

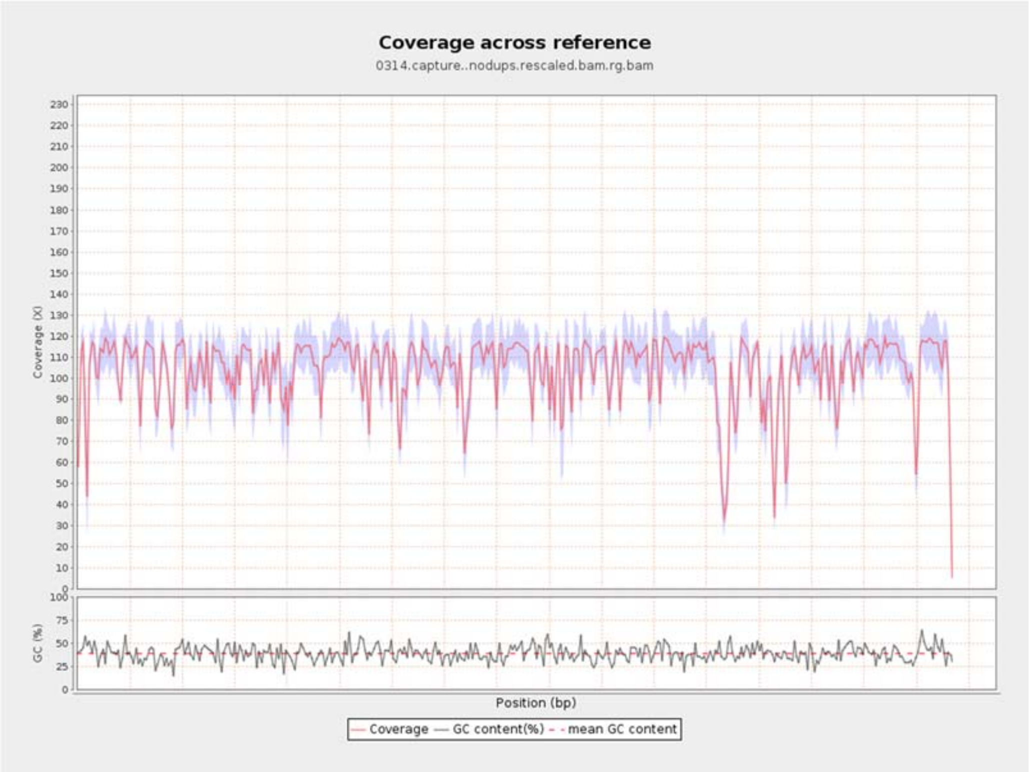

Supplementary Figure 19 | Coverage across the reference. Captured coat library (O314).
